# Sleep, Sensory Integration/Processing, and Autism: A Scoping Review

**DOI:** 10.3389/fpsyg.2022.877527

**Published:** 2022-05-17

**Authors:** Shelly J. Lane, Marco A. Leão, Virginia Spielmann

**Affiliations:** ^1^Sensory Integration, Play, and Occupational Therapy Research Lab, Department of Occupational Therapy, Colorado State University, Fort Collins, CO, United States; ^2^STAR Institute for Sensory Processing, Centennial, CO, United States

**Keywords:** autism spectrum disorder, sleep disturbances, sensory processing/integration, sensory reactivity, children, adults, insomnia

## Abstract

The prevalence of sleep dysfunction is considerably higher in the autistic population than in the non-autistic. Similarly, the incidence of sensory reactivity differences in autism exceeds that in the neurotypical population. The basis of sleep disorders in autism is multifactorial, but sensory integration/processing concerns may play a role. Research that investigates this interplay for autistic individuals is limited but vital. In this scoping review, we examined literature addressing the following research question: *What is the relationship between sleep and sensory integration/processing in autism?* We included articles if they were peer-reviewed, English or Spanish, purposefully addressed sensory integration/processing differences, were sleep focused and included autism as the primary diagnosis or population. Articles were excluded if the language was not English or Spanish, research was conducted with animals, they were non-peer-reviewed, the primary population was not autistic, the sensory focus reflected a specific sensorineural loss (e.g., blindness, or deafness), there was not a clear inclusion of sensory integration/processing or sleep. We searched six databases and included all citations from the inception of each database through June 2021. The search strategy identified 397 documents that were reduced to 24 included articles after exclusion criteria were applied. The majority of studies we identified characterized the relation between sleep and sensory integration/processing differences in autism. Investigators found multiple sleep concerns such as bedtime resistance, sleep anxiety, delayed sleep onset, night awaking, and short sleep duration in autistic individuals. Identified sensory concerns focused on reactivity, finding hyper- and hypo-reactivity as well as sensory seeking across sensory domains. Co-existence of sleep concerns and sensory integration/processing differences was frequently reported. Few intervention studies showed a clear sensory focus; those that did emphasized pressure, movement, touch, and individual sensory preferences/needs. Swimming programs and massage showed promising results. No studies were of high quality. At a minimum, there is a co-existence of sensory reactivity differences and sleep concerns in autistic children, and possibly autistic adults. The relationship between poor sleep and sensory integration/processing differences is complex and multi-faceted, requiring additional research. Interventions that purposefully include a central sensory component have not been well studied in autistic children or adults. Overall studies with greater rigor and purposeful use of sensation and sensorimotor supports as a component of intervention are needed. This study was not funded.

## Introduction

Sleep is a critical occupation for adequate neural function and maturation. Inadequate sleep has been linked to disruptions in attention, memory, mood, and behavior (cf. Souders et al., 2017; Tester and Foss, 2018), all of which influence participation across occupations. While many neurotypical children experience sleep difficulties, the incidence in autistic children^1^ is reported to be substantially higher; sleep disorders have been identified in as many as 50% of autistic adults and 80% of autistic children (Hirata et al., 2016; Souders et al., 2017; Deliens and Peigneux, 2019; Hohn et al., 2019). Reported sleep difficulties vary and may differ across the life span. However, the most often reported sleep concerns include reduced total sleep time, prolonged sleep latency, poor sleep efficiency, and wake after sleep onset; the literature is highly variable in both incidence and characteristics of sleep concerns (Malow et al., 2006; Miano et al., 2007; Goldman et al., 2012; Morgan et al., 2020).

Sensory reactivity differences are ubiquitous in autism and include sensory hyper-reactivity, sensory hypo-reactivity, and unusual sensory interests (Tomchek and Dunn, 2007; Crane et al., 2009; Marco et al., 2011; Puts et al., 2014; Tavassoli et al., 2014b; Taylor et al., 2020). Unusual sensory interests may be expressed as sensory seeking or craving (such as extensive smelling or touching of objects) and, along with reactivity differences, may be seen across the range of sensory domains (Schaaf and Lane, 2015). Importantly, investigators have indicated that sensory differences can negatively impact participation in autistic children (Little et al., 2015) and adults (Tavassoli et al., 2014b; Robertson and Simmons, 2015; Clince et al., 2016; Syu and Lin, 2018). More specifically, sensory integration/processing differences in autistic children have been associated with participation differences during mealtimes (Zobel-Lachiusa et al., 2015), in the classroom (Ashburner et al., 2008), during sleep (Reynolds et al., 2011), and with social participation (Watson et al., 2011). In addition, researchers such as Hochhauser and Engel-Yeger (2010) indicate that the choice of and setting for engagement in leisure is influenced by sensory seeking and sensory sensitivity. In autistic adults, sensory integration/processing differences have been shown to impact participation in higher education, and at least indirectly, social interactions (Robertson and Simmons, 2015; Syu and Lin, 2018), and other daily life activities (Tavassoli et al., 2014b). While there has been a great deal of research conducted on many of these associated occupational divergences, particularly in autistic children, the relationship between sensory integration/processing differences and the occupation of sleep has received only limited attention.

Importantly, both sensory reactivity differences and sleep concerns interfere with occupation and participation in activities of daily life (Roley et al., 2015; Dunn et al., 2016; Medic et al., 2017; Silverman and Tyszka, 2017; Berkley, 2021; Neufield et al., 2021). Clinically, understanding the range of these differences, as well as their inter-relatedness, has the potential to support more focused intervention. Deliens and Peigneux (2019) suggested that sensory reactivity and unusual sensory interests might play a role in sleep disturbances in autistic individuals. Drawing from the literature on neurotypical children (Tzchishinsky et al., 2008; Shochat et al., 2009), Deliens and Peigneux (2019) suggested that difficulty in the ability to filter out environmental sensation could interfere with sleep. Consistent with this suggestion, Hollway et al. (2013) indicated that to fall asleep and stay asleep individuals must be able to filter out sensation from the environment; as sensory reactivity differences are core to autism, this ability is heavily implicated.

Endeavoring to explain the link between historically perceived *core characteristics^2^* associated with autism (e.g., differences in social communication challenges, insistence on sameness, and resistance to change) and sleep difficulties, Hollway et al. (2013) suggested that autistic children may interpret external cues around bedtime to be stressors, leading to sleep difficulties. These bedtime challenges may result in hyperarousal and difficulty falling asleep (Deliens and Peigneux, 2019). Hollway and colleagues also indicate that the relationship between sleep challenges and autism may be bidirectional such that poor sleep exacerbates *features of autism*, which in turn leads to sleep challenges. Other investigators (Schreck et al., 2004; Hundley et al., 2016; Cohen et al., 2018) have also indicated that poor sleepers have more significant *autism features*. However, the bidirectional relationship between sleep and *features of autism* is not universally accepted (Deliens and Peigneux, 2019). For instance, and admittedly with some inconsistency, investigators have found that sleep problems in individuals with a range of *autism features* correlate with IQ (Gabriels et al., 2005; Bruni et al., 2007; Giannotti et al., 2008), and *challenging behaviors* in both children and adults (Limoges et al., 2005; Allik et al., 2006; Bruni et al., 2007). In addition, Hundley et al. (2016) indicated that sleep challenges do not correlate universally with what are considered *challenging behaviors.* Instead, they found that poor sleep correlates with high rates of repetitive sensory-motor behaviors but not insistence-on-sameness. These investigators indicated that intervention for the myriad sleep challenges needs to be multifaceted and should consider sensory aspects of the environment (Hundley et al., 2016).

We are faced with challenges. First, there is inconsistency in research relative to the relationship between sleep difficulties and sensory integration/processing differences. Second, there remains some uncertainty about the impact of poor sleep and sensory integration/processing differences on occupation and participation in autistic individuals. Together these factors make for challenges in appreciating this interplay and lead to difficulties in providing appropriate intervention services. Further, investigations looking at this interconnectedness focus primarily on children with very limited information available for autistic adults. In this review, we examined how this relationship is characterized and defined by current research and looked at the interventions that have been used. Due to the broad nature of this aim, we determined that a scoping review was the most appropriate approach.

In conducting our review we considered both the National Institute of Mental Health Research Domain Criteria (RDoC) framework^3^ and the Diagnostic and Statistics Manual V (DSM-V; American Psychiatric Association [APA], 2013) to examine the interplay between the dimensions of sleep and sensory integration/processing. The RDoC is a framework designed to guide understanding of mental health as well as illness, in psychological and biological systems. The RDoC addresses the construct of Sleep/Wakefulness within the domain of Arousal and Regulatory Systems. Sensory reactivity, of interest in this review, is arguably also included under arousal in the domain of Arousal and Regulatory Systems: ‘‘sensitivity of the organism to stimuli, both external and internal.’’^4^ Sensorimotor systems are represented as a separate domain and include constructs of motor actions, agency and ownership, habit–sensorimotor, and innate motor patterns. In addition, sensory systems are embedded within the Cognitive Systems domain under the construct of perception (visual, auditory, olfactory, somatosensory, and multimodal). While the inclusion of both sleep and sensory reactivity within the Arousal and Regulatory system domain supports our examination of this interaction, the multiple representations of sensory integration/processing across other domains present challenges for examining the interaction of these constructs. In addition, there have been notable calls to include sensory processing as a unique domain in the RDoC (Harrison et al., 2019), and leading theorists are recognizing the importance of sensory integration/processing across disciplines (Bogdashina, 2016; Robertson and Baron-Cohen, 2017; Sinclair et al., 2017; Mueller and Tronick, 2020). These positions closely support our perspective on the centrality of sensory integration/processing to the development of health, wellness, occupation, and participation, providing support for this review. Within the DSM-V the foundational importance of sensory integration/processing to wellbeing finds some additional support, although it comes from the perspective of dysfunction. Within this framework, sensory reactivity differences are associated with autistic *core characteristics* and included under the “restricted, repetitive patterns of behaviors, interests, or activities” diagnostic feature. While no direct link is made to sleep concerns, they are considered either a reflection of underlying anxiety or depression, or a concomitant *feature of autism*.

## Methods

This scoping review was structured based on the framework developed by Arksey and O’Malley (2005) and guidelines from the Joanna Briggs Institute (Peters et al., 2020), and utilized the PRISMA-ScR checklist (Tricco et al., 2018). This project was registered with Prospero, CRD42020209872.

### Identifying the Research Question

Our intention in this review was to examine literature that investigated the relations between sensory integration/processing differences and sleep challenges in autistic individuals, across the life span. Our research question was: *What is the relationship between sleep and sensory integration/processing in autism?*

### Identifying and Locating Relevant Studies

We conducted an initial search in CINAHL, Pubmed, PsychINFO, Academic Search Premier, Web of Science, and Embase databases, and included all citations from the inception of each database through November 2020. A sample search strategy comprised the terms: [sleep OR “sleep-wake disorders” OR “sleep dysfunction” OR “sleep disorder” OR “sleep problems” for Sleep; [“sensory processing” OR “sensory processing disorder*” OR “sensory integration” OR “sensory integration dysfunction*” OR “sensory integration dysfunction” OR “sensory processing disorder” OR “sensation disorder” OR “sensory over responsivity” OR “sensory reactivity” OR vestibular OR propriocept* OR interocept* OR tactile OR touch OR somatosensory OR “somatosensory” OR Postur* OR “multi sensory” OR multisensory OR sensorimotor] for sensory integration/processing; and [“autism spectrum disorder” OR “autistic disorder” OR asperger OR autism OR autistic] for autism. The search terms were entered into the databases with an “AND” term between each of them.

In June 2021, we conducted a follow-up search in the same databases to check for newer articles. We hand searched the reference lists from included articles (August 2021) to ensure that all appropriate articles were comprised.

### Study Selection

Based on our research question, we set the inclusion criteria to be as follows: (1) peer-reviewed articles (qualitative or quantitative research papers), written in English or Spanish; (2) sensory integration/processing differences purposefully included (3); sleep focused and (4) autism as the primary diagnosis or population. We detailed the exclusion criteria in the following hierarchical order: (1) articles written in a language other than English or Spanish; (2) articles conducted with animals; (3) presentations, conference proceedings, non–peer-reviewed research literature, dissertations, and theses; (4) primary focus on populations other than autism; (5) sensory focus on a specific sensorineural loss (e.g., blindness, or deafness); (6) no clear inclusion of sensory integration/processing or sleep.

**FIGURE 1:**
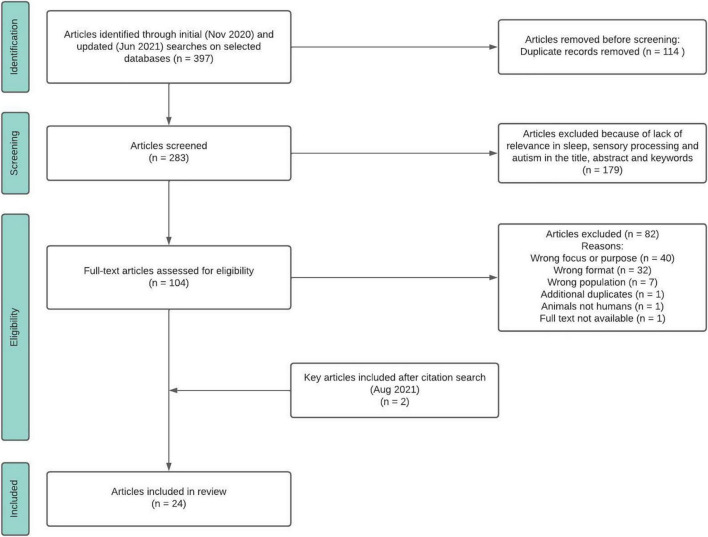
Preferred Reporting Items for Systematic Reviews and Meta-Analyses (PRISMA- ScR).

Initial and follow-up searches resulted in a total of 397 references. Of these, 114 duplicate articles were removed. A total of 283 abstracts were screened by title and abstract by two reviewers; conflicts were resolved by a third reviewer or through discussion and 179 articles were excluded. Two reviewers conducted full-text reviews of the remaining 104 potential articles; 82 additional articles were excluded for the following reasons: one study was conducted with animals rather than humans; 32 articles were not peer reviewed, seven articles collected data from populations other than autism; 40 studies did not include a clear definition of sensory integration/processing or sleep; one article was an additional duplicate and one full text was not available. Per hand search of reference lists in all included articles, we identified and added two additional articles (Table 1). Figure 1 shows the Preferred Reporting Items for Systematic Reviews and Meta-Analyses (PRISMA-ScR; Tricco et al., 2018) flow diagram of the search strategy. The search strategy used for Web of Science is available as a Supplementary Table.

**TABLE 1:** Results per database.

		Results
Pubmed		85
EBSCO Host	CINAHL	33
	PsychINFO	52
	Academic search premier	32
Web of science		87
Embase		108
Hand search		2
Total		399

### Charting the Data

A total of 24 articles met the inclusion criteria and were included in the final review (Figure 1). Data extraction was conducted using these fields: title, authors, year, journal, source country, study design, research question, sample size, and characteristics, inclusion and exclusion criteria, diagnostic tools, measures of sensory integration/processing, sleep and other characteristics, intervention, quantitative and qualitative findings, identified relationships between sensory integration/processing and sleep, and authors conclusions.

### Collating, Summarizing, Reporting

We collated and summarized the data from the extraction table and determined there were two broad categories into which studies fell: characterization and intervention. We present results based on these categories.

## Results

A total of 24 articles were included in the data extraction. Of these, 17 articles offered information characterizing a relation between sleep concerns and sensory integration/processing differences (Table 2), and seven articles were intervention studies using approaches with a clear sensory focus (Table 3). We considered these interventions *sensory-based* if consideration of the sensory components was a focal point, or *sensory incidental* when the intervention provided sensory input, but the inclusion of sensation was not the primary focus.

**TABLE 2:** Characterizing sleep/sensory processing relations.

References, table, ID#	Study design and aim	Participants, *N*, Age range, M_*age*_, % Males	Sensory processing tool; areas of sensory difference	Sleep tool and areas of difference	Other measures related to aims	Findings related to sleep and sensory processing	Country	Quant	Qual
Eyuboglu and Eyuboglu, 2020, #2	Cross-sectional, descriptive, correlational Aim: Examine incidence of sleep problems in autistic vs NT children; examine the relationship between maternal anxiety and child sensory reactivity and sleep problems.	ASC *N* = 48, age 18-60 months M_*age*_ = 33.3 + 9.6 83% male; NT *N* = 51, 18-60 months**	Sensory reactivity scale (purpose built): autistic children had higher scores in SOR, SUR, SS	CSHQ: autistic children had higher frequency of BR; TST; Parasomnias; amount of sleep	Hospital anxiety depression scale (mother); AuBC; CARS	Sleep concerns and sensory reactivity differences more prevalent in autistic children; autism severity correlated with sensory reactivity and sleep concerns; maternal depression and anxiety correlated with sleep problems and sensory reactivity; parasomnia predicted maternal depression	Turkey	X	X

Ghanbari and Rezaei, 2016, #6	Cross-sectional description, correlational Aim: Determine relationship between sensory processing disorder and sleep disturbance in autistic children	ASC *N* = 35, age 3-12 years M_*age*_ = 9 + 2.30 80% male	SSP: 95.3% showed some degree of sensory processing disorder	SDSC: 68.6% showed sleep disturbances	Demographic form	No significant relationship between sensory processing disorders and sleep disturbances	Iran	X	

Hohn et al., 2019, #8	Cross-sectional, descriptive, correlational Aim: Examine link between sensory responsiveness, social skills, and insomnia in autistic adults	ASC *N* = 631 18-65 years M_*age*_ = 42.62 + 12.21 48% male	SPQ, Short Form: outcome not specified	ISI: Subthreshold insomnia, higher than reported in general population; influenced by sex, IQ	Autism Spectrum Quotient-28 Social Skills Subscale	Insomnia extends into adulthood for autistics; severity of insomnia symptoms predicted by high levels of sensory reactivity and lower social skills. Sensory reactivity impact seems driven by visual system	Netherlands	X	

Hollway et al., 2013, #9	Cross-sectional retrospective, correlational chart analysis Aim: Explore variables related to sleep in autistic children to replicate prior findings; provide foundation for evidence-based interventions.	ASC *N* = 1583 2-17 years M_*age*_ = 6.34 ± 3.5 84% male	SSP: Domain findings not presented	CSHQ: Domain findings not presented	VABS, MSEL, SB5, CBCL	Greater taste/smell impairment associated with more sleep anxiety; greater SUR, SS, and auditory filtering contributed to a prediction of CSHQ 23-item total score***	United States	X	

Jamioł-Milc et al., 2021, #10	Cross-sectional, descriptive, correlational Aim: Determine whether Tactile Stimilation Modulation disorders are linked to insomnia in autistic children.	ASC *N* = 27 M_*age*_ = 6.8 ± 2.9 years** 81.5% male	Identification of TSM disorder via parent interview; observation of tactile responsivity according to behaviors identified by Miller, et al: Tactile SOR 74.1%; tactile SUR 25.9%	AIS (difficulty falling asleep, night and early morning awakenings, TST, and wellbeing during the next day): 40.7% children showed insomnia	Purpose built parent questionnaire (pregnancy, childbirth, perinatal circumstances, school history, sensitivity towards tactile stimuli at home)	Trend toward higher prevalence of insomnia in autistic children with tactile SUR; lack of significance related to small sample size	Poland	X	

Klintwall et al., 2011, #11	Cross-sectional, descriptive, correlational with ASC subgroups Aim: Describe sensory differences in preschool autistic children; compare autism across autism subgroups; relate findings to other clinically relevant symptom domains	ASC *N* = 208 <4.5 years** 84.6% male	PARIS schedule interview (SOR,SUR): > 1 major sensory difference found for 76% children: SOR: 44% sound, 19% touch, 5% smell; 19% visual stimuli; SUR: 40% pain, 22% cold, 7% heat. Number of sensory differences varied across autistic subgroups	None specified	AuBC; VABS; cognition; expressive language	Greater number of sensory differences found in autistic children with sleep problems	Sweden	X	

Kosaka et al., 2021, #12	Two-group comparison, cross-sectional, descriptive, correlational Aim: Validate relationship between sensory characteristics and sleep dynamics among autistic children	ASC *N* = 20, 3-6 years M_*age*_ = 5.1 + 1.3 85% male; NT *N* = 20 3-6 years M_*age*_ = 5.1 ± 0.9 years 60% male	SP-J; all sensory subscales differed between groups for both high and low threshold items	JSQP: autistic children showed higher scores RLS, SOSA; CRD; DS, SE, WASO, TST parasomnias, insomnia Actiwatch Spectrum Plus: significant group difference in activity during sleep, activity per minute during sleep	NA	Activity per minute during sleep in autistic group correlated with vestibular and oral sensory sensitivity	Japan	X	

Manelis-Baram et al., 2021, #14	Longitudinal, descriptive, correlational Aim: Further examine the longitudinal relationship between sleep disturbances and sensory sensitivities	ASC *N* = 103 M_*age*_ = 3 + 1.12 years [at baseline, T1]**, M_*age*_ = 4.5 + 1.19 years [at follow-up, T2]** 75.7% male	Infant/Child SP: All children showed SOR (avoiding and sensitivity), SUR, SS above expected levels at baseline	Hebrew CSHQ autistic children showed BR, SOD, SD, SA, NW, SDB, DS, parasomnias, children sleeping 1-2hr 35min less than NT peers	BSID WPPSI	Changes T1 to T2: 35% children had worse sleep, 34% were stable; 28% had greater sensory sensitivity, 55% stable; 34% had more sensory avoiding, 43% stable; 34% had more SS, 49% stable; 23% had worse sensory registration, 52% stable. Changes in sleep paralleled sensory changes, except for sensory seeking. Significant total sleep disturbance correlated with sensory sensitivity, sensory avoiding, and sensory registration, not with SS. Changes in sleep disturbances correlated with sensory sensitivity only. Regression showed sleep T1 predicted sleepT2, and sensory sensitivity was only sensory quadrant that improved prediction.	Israel	X	

Mazurek and Petroski, 2015, #15	Cross-sectional, descriptive, correlational Aim: Determine the relationships among sleep problems, sensory problems, and anxiety in autistic children.	ASC *N* = 1547 2-17.6 years; M_*age*_ = 7.9+3.4 younger group 2-5 years** 82.9% male; older group 6-18 years** 85.9% male	Subset of SSP reflecting SOR in touch, taste/smell, movement, visual and auditory domain; Domain findings not presented	CSHQ: Domain findings not presented	CBCL DSM-oriented Anxiety Problems scale	For both age groups, SOR correlated with all subscales of CSHQ using bivariate model Multivariate model: younger children showed SOR associated with SOD, SD, NW, but no other sleep challenges; older children showed links between SOR and all sleep challenges other than NW. Anxiety showed bivariate and multivariate relationship with all sleep challenges for both groups	United States	X	

Mazurek et al., 2019, #16	Descriptive, correlational, longitudinal Aim: Examine chronicity of sleep disturbance in autistic children; determine longitudinal relations among sleep problems and co-occurring symptoms	ASC *N* = 437 younger group 2-3 years [at T1] M_*age*_ = 2.98 +.58 84% male; older group 4-10 years [at T1]; M_*age*_ = 6.35 +1.7 81.5% male	Subset of SSP reflecting SOR in touch, taste/smell, movement, visual and auditory domain: SOR	CSHQ: BR; SOD; NW; SDB; SD; SA;DS; TST	CBCL scales: Aggressive Behavior Syndrome Scale; Attention Deficit/Hyperactivity DSM-Oriented Scale; Anxiety Problems DSM-Oriented Scale; Somatic Complaints Syndrome Scale; ABC; AuBC	Significant relationship between sleep difficulties and SOR in both younger and older participants; SOR predicted later sleep problems in younger children, and sleep disturbance longitudinally predicts hyperactivity and attention challenges	United States	X	

Nieminen-von Wendt et al., 2005, #17	Cross-sectional, descriptive, correlational Aim: Determine if a set of clinical features not included in the DSM-IV or ICD-10 for AS, are associated with AS or a familial trait that is not related to AS.	*N* = 10 families (138 individuals), 58 ASC 4.5-78.2 years M_*age*_ = 32.8	Purpose built questionnaire (face recognition difficulties, presence of aberrant sensibilities, aberrant eating habits): Face recognition differences and aberrant sensitibilities (touch, light, sound, smell) high in family members with AS; aberrant eating habits higher in AS	Purpose built questionnaire (sleeping disturbances): Sleep disturbances in AS, 48.3%; NT 23.2% Risk of sleep problems slightly higher in AS	VABS	No clear links examined; both differences in sensory processing and greater sleep disturbances are important to consider in AS.	Finland	X	

Ornitz et al., 1973, #18	Cross-sectional, descriptive, correlational Aim: Examine sleep characteristics of autistic and NT children under 3 sensory conditions during sleep: no stimulation; mild continuous sinusoidal vestibular stimulation; auditory click stimulation	ASC *N* = 6 39.3-94.5 months M_*age*_ = 57.57 100% male; NT *N* = 8 43-128 months M_*age*_ = 65.44 75% male	NA	REM activity during REM sleep; # REM periods, duration of REM burst within each REM period, duration of REM period, % time in REM, # night wakings; proportion of time awake.	EEG to determine sleep stage; eye movements to reflect REM bursts	Autistic children showed fewer REM burst eye movements with vestibular stimulation, suggesting under-responsivity	United States	X	

Reynolds et al., 2012, #19	Cross-sectional, descriptive, correlational Aim: Examine relationship between physiologic responses to sensation and sleep in autistic and NT children; determine variables distinguishing good from poor sleepers	ASC *N* = 27 6-12 years M_*age*_ = 104.85 + 21.9 85% male; NT *N* = 29 6-12 years M_*age*_ = 105.04 + 22.9 50% male	SP: ASC children had higher percentage of definite difference scores for all SP quadrants, significantly greater dysfunction relative to NT children; 81% scored definite difference for at least one quadrant	Sleep questions from CBCL formed sleep index (nightmares, overtired, sleeps less than most kids, sleeps more than most kids, talks or walks in sleep, trouble sleeping): autistic children had higher frequency of problem sleep behaviors; sleep index in autistic children higher	EDA, EDR, cortisol response to sensory challenge	Autistic children have more sleep disturbances and sensory modulation differences than NT; sensation avoiding highly correlated with sleep problems; poor sleepers together had have high afternoon cortisol, greater EDR response to sensory challenge, trend toward higher cortisol 25-30 minutes post sensory challenge; auditory stimulus most salient in distinguishing good vs poor sleepers; poor sleepers also had higher magnitude responses to smell and visual stimuli	United States	X	

Sacco et al., 2010, #20	Cross-sectional, descriptive, correlational Aim: Delineate medical components of autism; assess association with biological endophenotypes	ASC *N* = 245 2-30 years M_*age*_ = 8.82 + 5.62 88.2% male	Purpose built questionnaire (pain sensitivity, presence of muscle hypotonia at intake): Decreased pain sensitivity, 36.7% Hypotonia, 10.2%	Purpose built questionnaire (sleep disorder at intake): Not specified	VABS, Griffith Mental Developmental Scales; Colored Raven Matrices; Bayley Developmental Scales; Leiter International Performance Scale	Identified four medical components of autism, one termed ‘circadian and sensory dysfunction’	Italy	X	

Silva and Schalock, 2012, #21	Cross-sectional, descriptive, measure validation Aim: Validation of Sense and Self-regulation Checklist (SSC).	ASC *N* = 99 M_*age*_ 3.9 + 1.2** 81.8% male DD *N* = 28 M_*age*_ = 2.26 + 1.4** 60.7% males NT *N* = 138 M_*age*_ = 3.9 +.89** 50.7% male	SSC: SOR and SUR in 4 sensory domains (touch–pain auditory, visual, taste–smell) broken into body part areas: 96% prevalence of ≥ 1 SOR or SUR response; more SOR than SUR	SSC, self-regulatory items re sleep: Significant differences for autistic children relative to others for TST, and total sensory + TST; all self-regulatory domains differentiated autistic children from others	PDDBI	Self regulatory and sensory differences co-exist significantly; sleep is negatively impacted	United States	X	X

Tzischinsky et al., 2018, #23	Two-group comparison; correlational Aim: Perform more in depth study of sleep disturbances and sensory differences in children with autism	ASC *N* = 69 3-7 years M_*age*_ = 4.94+1.23 81% male NT *N* = 62 3-7 years M_*age*_ = 4.84 + 1.15 66% male	SP: significant between group differences in all 5 sensory modalities; autism group significantly lower scores compared to norms and controls. Autism group had lower scores for both high and low threshold items.	CSHQ: Autism group had greater disturbance for total score and all subscales except sleep DB, NW, DS		Autism group showed significant negative correlation between touch + oral sensitivity and total sleep disturbance; control group between touch + vestibular sensitivity and total sleep disturbance. Both groups showed same pattern of scores for low threshold items. Touch sensitivity predicted 29% of variance in total sleep disturbance score in autism group, 16% in control; 20% of variance in controls explained by vestibular sensitivity. Touch low threshold items alone predicted 24% variance in total sleep disturbance in autism group.	Israel	X	

Wang et al., 2019, #24	One-group, cohort, cross-sectional Aim: Evaluate association between sensory processing problems and sleep disturbances, emotional and behavioral problems and abnormal mealtime behaviors in autistic children	ASC *N* = 81 3-6 years M_*age*_ = 5.18 +.92 82.7% male NT *N* = 153 3-6 years M_*age*_ = 5.34 + 1.14 73.2% male	SSP: ASC significantly more SUR, SS, auditory filtering, low energy/weak, and total	C-SHQ: Greater BR, SOD, SA, DS, total sleep score	SCQ, SDQ, Mealtime Behavior Questionnaire; Peabody Picture Vocabulary Test	In autism group, significant relationship between CSHQ total and SOR for tactile and movement, SUR, SS, low energy/weak, total SSP; SUR and SS explain 8% variance in total CSHQ scores; SSP total explained 18.7% variance in sleep disturbance	China	X	

*Descriptive terms: AS: Asperger Syndrome; DD: developmental disability; NT: neurotypical; LD: learning disability.*

*Assessment tools: ABC: Aberrant Behavior Checklist; AIS: Athens Insomnia Scale AuBC: Autism Behavior Checklist; BSID: Bayley Scales Of Infant and Toddler Development; CARS: Childhood Autism Rating Scale; CBCL: Child Behavior Checklist; CSHQ: Children’s Sleep Habits Questionnaire; C-SHQ: Chinese Sleep Habits Questionnaire; EDA: electrodermal activity EDR: electrodermal response; ISI: Insomnia Severity Index; JSQP: Japanese Sleep Questionnaire for Preschoolers; MSEL: Mullen Scales of Early Learning; PDDBI: Pervasive Developmental Disorders Behavior Inventory; SB5: Standford Binet 5^th^ edition; SCQ: Social Communicatoin Questionnaire; SDQ: Strengths and Difficulties Questionnaire; SDSC: Sleep Disturbance Scale for Children; SP: Sensory Profile; SP-J: Japanese Sensory Profile; SPQ: Sensory Perception Quotient; SSP: Short Sensory Profile; TMS: Tactile Stimulation Modulation; VABS: Vineland Adaptive Behavior Scale; WPPSI: Wechsler Preschool and Primary Scale of Intelligence.*

*Sleep parameters: BR: bedtime resistance; CRD: circadian rhythm disorder; DS: daytime sleepiness; RLS: restless leg syndrome; SA: sleep anxiety; SD: sleep duration; SDB: sleep disordered breathing; SE: sleep efficiency; SOD: sleep onset delay; SOSA: sensory obstructive sleep apnea; NW: Night Wakings; TST: total sleep time; WASO: wake after sleep onset.*

*Sensory domains: SOR: sensory over- reactivity or responsivity; SS: sensory seeking; SUR: sensory under-reactivity or responsivity.*

* Other details on age not provided.

^**^These articles appear to be duplicates in terms of participants, design, and outcomes; different authors and journal. Only one was fully reported?in the current review.

^***^CSHQ Total Score was based only on items listed in insomnia subscale.

**TABLE 3:** Interventions addressing sleep and sensory processing.

References, table 3 ID#	Study aim(s) and design	Participants, age range, % male	Sensory processing tool; areas of sensory difference	Sleep tool and areas of difference	Other measures	Intervention	Findings	Country	Quant	Qual
Cullen et al., 2005, #1	One-group cohort, exploratory Aim: Explore experience of parent/child touch before and after touch therapy training program; develop model of touch therapy process	ASC *N* = 14 2-13 years** 87% male	Parent report of tactile defensiveness, food aversion related to texture and temperature	Parent report of poor sleep patterns	Parent interview and Home Record Sheet		Improved sleep patterns in 6/7 children with sleep difficulties; more relaxed and calmer child; improved tolerance of touch, increased sense of closeness between parent and child	UK		X

Gee et al., 2021a, #3* (Gee et al., 2021b, #4*)	Single subject, pre-post test ABA Aim: Assess effectiveness of weighted blanket use on sleep quality in autistic children with sleep disturbances and SOR	*N* = 2 P1: 4 years 5 months, male; P2: 4 years 1 month, female	SPM-P: P1: definite difference for tactile, auditory, visual SOR P2: definite difference for tactile, auditory, visual SOR	CHSQ; Sens Sleep App P1: poor sleep quality; difficulty falling asleep (seven days a week), WASO (seven days a week), wakes up too early (five days a week), experiences a poor morning mood (five days a week) P2: difficulty staying asleep (wakes > x1, five days a week), wakes up too early (seven days a week), experiences a poor morning mood (five days a week).	Daily online survey re sleep parameters	A(1): 9 dy baseline; B: 14 consecutive days weighted blanket (10% body weight) use; A(2): 7 days, no blanket. Data collected for time to fall asleep, number of wakings, hours of sleep, morning mood	Weighted blanket had little influence re improving sleep quality through the objective and subjective measures	United States	X	

Gee et al., 2016, #5	Single subject with repetition; ABA design Aim: Explore effectiveness of weighted blankets with autistic children ages 3-6, and SOR (touch and/or auditory)	*N* = 2, P1: 4 years, 2 month, male; P2: 5years, 1 month, male	SPM: P1: definite difference in social participation, visual, auditory, tactile processing, body awareness, balance and motion, planning and ideas P2: definite difference in social participation, visual, auditory, tactile processing, body awareness, balance and motion, planning and ideas	CHSQ; P1: falling asleep on his own, NW, staying in bed at bedtime, SD P2: WASO, fear of the dark, breathing difficulties (chronic congestion, history of ear infections) at night, awakening in negative mood	Daily online survey re sleep parameters	A(1): 9 days, baseline; B: 14 consecutive days weighted blanket (10% body weight); A(2): 7 days, no blanket. Data collected for time to fall asleep, number NW, hours of sleep, morning mood	Minimal improvement (slight increase in TST/night and decrease in time to fall asleep).	United States	X	

Gringras et al., 2014, #7	RCT, multicenter, controlled, crossover Aim: Determine if weighted blankets increase TST and improve other sleep parameters for autistic children	*N* = 73; 6 discontinued 5-16 years M_*age*_ = 8.7 + 3.3 88% male [in intervention first], M_*age*_ = 9.9 + 2.8 74% male [in control first]	SSP: domain scores not provided	Baseline parent report: Failing to fall asleep within 1 hr of “lights off”, 3/5 and/or failing to achieve 7 hrs continuous sleep, 3/5 nights. Study measures: actigraph and sleep diary for TST, SOL, CHSQ: SOD, poor sleep maintenance, poor sleep onset and maintenance	CSDI, ABC, SBQ, SCQ	Baseline: 7-21 dys; Weighted blanket vs non-weighted blanket, 12-16 dys	No difference in TST, SE, WASO, sleep latency between blankets in actigraph or sleep diary. CSDI showed slight improved sleep with control blanket. Children “really liked” weighted blanket more than control; parents indicated sleep was much better, child calmer with weighted blanket.	UK	X	X

Lawson and Little, 2017, #13	One-group, cohort, cross-sectional, pre-post test Aim: Understand effects of swimming on sleep in ASC children Examine feasibility of swimming program Define features of children showing decreased sleep disturbance	ASC *N* = 10 5-12.3 years M_*age*_ = 7.5 + 2.4 years	SP: Definite or probable difference for all quadrants for both responders and non-responders	CSHQ: Elevated sleep disturbance scores at baseline	Demographic form SRS Parent satisfaction questionnaire	8 wkly 30-minute swim lessons; 1:1 with social opportunities (e.g., songs and games) at the start/end each lesson. Lessons individualized based on learning and sensory preferences, emphasized both skill development and water safety	All families completed; high parent satisfaction; intervention feasible. Variable changes in sleep (4/10 improved, 1/10 remained the same; 5/10 increased sleep disturbance). Responders were older, had decreased ASC severity, attended more sessions and had sensory characteristics reflecting high sensory sensitivity and avoidance, with low SS	United States	X	X

Silva et al., 2007, #22	One-group, cohort, cross-sectional, pre-post test Aim: Replicate earlier study with small controlled sample and blinded examiners	ASC *N* = 15 [intervention] *N* = 7 [control] 3-6 years M_*age*_ = 3.9 + 1.2 81.8% male At 5 months control group also received treatment; *N* = 5	SP; All five senses were involved, although different children had different combinations of involvement Response to massage tool; scoring number of areas of aversion and duration of tolerance to touch. Video of first visit:	Parent questionnaire sleep items	BDI: Cognitive Domain Screening Test VABS: Daily Living Skills, Socialization, Communication, Motor domains Parent Questionnaire addressing bowel patterns Study designed Scoring Tool for Cignolini Method	The Cignolini Qigong methodology: 11 different Qigong massage movements from head to foot along acupuncture channels; duration of 15 min. Delivered for 5 months total, alternating practitioner administration twice daily for 5 weeks with parent administration at least once daily for 5 weeks.	SP scores improved overall (total SP) and within each sensory domain in intervention group; decrease in number of body areas showing adverse responses to gentle touch; improved sleep and bowel concerns. Improvements in daily living skills and social learning	United States	X	

*Descriptive terms: NT: neurotypical; STS: sleep to sound mattress.*

*Assessment tools: ABC: Aberrant Behavior Checklist; BDI: Batelle Developmental Inventory; CCC: Children’s Communication checklist; CFQL: Child and Family Quality of Life questionnaire; CSDI: Composite Sleep Disturbance Index; CSHQ: Children’s Sleep Habits Questionnaire; FISH: Family Inventory of Sleep Habits; PDDBI: Pervasive Developmental Disorders Behavior Inventory; SBQ: Sensory Behavior Questionnaire; P: Sensory Profile; SCQ: Social Communication Questionnaire; SRS: Social Responsiveness Scale; SSP: Short Sensory Profile; VABS: Vineland Adaptive Behavior Scale.*

*Sleep parameters: DS: daytime sleepiness; SD: sleep duration; SOD: sleep onset delay; SOL: sleep onset latency; NW: Night Wakings; TST: total sleep time; WASO: wake after sleep onset.*

*Sensory domains: SOR: sensory over- reactivity or responsivity; SS: sensory seeking; SUR: sensory under-reactivity or responsivity.*

*These articles appear to be duplicates in terms of participants, design, and outcomes; different authors and journal. Only one was fully reported in the current paper.

^**^ Other details on age not provided.

### Measures of Sleep and Identified Concerns

The most commonly reported tool used to reflect sleep concerns was the *Children’s Sleep Habits Questionnaire* (CSHQ; Owens et al., 2000). This screening tool asks parents to reflect on their child’s sleep characteristics over a typical recent week. Subscales include bedtime resistance, sleep onset delay, sleep duration, sleep anxiety, night wakings, sleep disorder breathing, parasomnias, and daytime sleepiness. A total sleep score is also generated (Owens et al., 2000). The original version of this tool had 45 questions [CSHQ (45)]; the authors created a 33-item revised version to reduce redundancy and ambiguity [CSHQ (33)]. The CSHQ, or a version of it, was used to define sleep concerns in seven of the 17 articles that characterized a relationship between poor sleep and sensory reactivity differences (Table 2, studies #2, #9,#14, #15, #16,#23,#24) and one of the seven articles that utilized sensory-based interventions (Table 3, study #13). A variety of other means of determining sleep concerns, including both published tools and those purpose-built for individual studies, were used by other authors (Tables 2, 3). In some studies, sleep concerns were identified using both subjective measures based on parent report, and more objective measures such as actigraph (Table 2, study #12; Table 3, study #7). Sleep concerns of autistic children were multifaceted and included concerns in all domains tapped by the CSHQ, along with wake after sleep onset. Hohn et al. (2019), the only study found addressing adults, reported that autistic adults experienced an elevated incidence of insomnia.

### Measures of Sensory Integration/Processing and Identified Concerns

Sensory integration/processing was assessed using a variety of tools, although some form of the *Sensory Profile* ([SP]; Dunn, 1999) or *Short Sensory Profile* ([SSP]; McIntosh et al., 1999) were used most commonly (Table 2, studies #6, #9, #12, #14, #15, #16, #19, #23, #24; Table 3, studies #7, #13, #22). In the SP and the SSP the authors consider the interface between neurological threshold and self-regulation in response to sensation, defining sensory processing patterns across four quadrants: poor sensory registration, sensory seeking, sensory avoiding, and sensory sensitivity. With the SP, Dunn also identifies reactivity differences within each sensory domain (sensory section scores) along a continuum from hyper- to hypo-reactivity, and within behavioral domains (behavioral section scores); this finer delineation is not available to users of the SSP. Thus, in these identified studies, the focus of sensory integration/processing differences was on sensory reactivity rather than perception or discrimination. There was variability in examining and reporting sensory differences across the studies. However, overall findings can be generalized to reflect sensory hyper-reactivity (which includes both sensory avoiding and sensory sensitivity), hypo-reactivity, and sensory seeking. Often a combination of these sensory processing differences was identified. While some investigators reported differences within specific sensory domains, we did not find consistency across studies; investigators variously reported sensory processing differences within visual, tactile, auditory, taste-smell, and vestibular sensory domains.

### Characterizing the Relation Between Sleep Disturbances and Sensory Integration/Processing Differences

Most investigators agreed that there was, at minimum, a co-existence of sensory reactivity differences and sleep concerns. This finding was clearly stated by Silva and Schalock (2012): sleep and sensory processing differences co-exist in autism, and disordered sensory processing has a negative impact on sleep. In a family-based study, Nieminen-von Wendt et al. (2005) set out to examine familial traits of Asperger Syndrome not included in specific diagnostic criteria (DSM-IV or ICD-10). While they did not delineate a specific relationship between sleep concerns and sensory differences, they did find that these concerns and differences co-existed in their participants. These investigators suggested that sensory processing differences might be considered in the diagnostic criteria for Asperger Syndrome. Other investigators indicated that the combination of sleep concerns and sensory reactivity differences in autistic children exceeded that found in neurotypical children (Nieminen-von Wendt et al., 2005; Reynolds et al., 2012; Tzischinsky et al., 2018; Wang et al., 2019; Eyuboglu and Eyuboglu, 2020) and that a greater number of sensory integration/processing differences could be seen in autistic children with sleep concerns (Klintwall et al., 2011). Jamioł-Milc et al. (2021), investigating tactile modulation differences in autistic children through parent interview and observation, identified a potential relationship between tactile hypo-responsivity and insomnia but no relation between tactile hyper-responsivity or sensory seeking and insomnia. Interestingly, Ghanbari and Rezaei (2016) found no relationship between sensory processing differences and sleep concerns.

Using a Hebrew version of the CSHQ (45) (Tzchishinsky et al., 2008) and a version of the Infant-Child Sensory Profile (Neuman et al., 2004; Dunn, 2014) that had been validated in Israel, Manelis-Baram et al. (2021) examined the relationship between sleep and sensory reactivity at ages 3 (time 1) and 4.47 (time 2) years. Looking first at time 1, investigators indicated that sensory sensitivity was the only reliable indicator of sleep disturbance when controlling for scores in other sensory quadrants on the SP. Sensory sensitivity showed a strong relationship with both nighttime and total sleep time (including naps). Looking at change over time, these investigators found that more than 50% of their participants showed considerable changes (positive or negative) in either sleep severity or sensory reactivity. Hierarchical regression modeling, using age at time 1, time between assessments, time 1 sleep score, and the change in sensory sensitivity between times 1 and 2, investigators strongly predicted sleep disturbance at time 2; no other measure of sensory reactivity contributed to this prediction. They concluded that sleep disturbance and sensory reactivity severity were coupled and possibly rooted in a common physiological mechanism.

With a somewhat different focus, Mazurek and Petroski (2015) and Mazurek et al. (2019) drew items from the SP that reflected only sensory hyper-reactivity. Mazurek and Petroski (2015) grouped children with autism into younger (2–5 yrs.) and older (6–18 yrs.) subsets and found sensory hyper-reactivity to significantly correlate with all CSHQ (45) subscales. Applying a path analysis, they found sensory hyper-reactivity to be associated only with sleep onset delay, sleep duration, and night waking for the younger group. In contrast, sensory hyper-reactivity was related to all CSHQ (45) subscales except night waking for the older children. In a later study, Mazurek et al. (2019) identified a relationship between sensory hyper-reactivity and all subscales of the CSHQ (45) in both preschool and school-aged children. These investigators further indicated that sensory hyper-reactivity in preschoolers predicted sleep challenges at school age.

Looking within specific sensory systems, Tzischinsky et al. (2018) used the Hebrew version of the SP (Neuman et al., 2004) and found that while tactile and oral sensitivity differences were related to total sleep disturbances [Hebrew CSHQ (45)], tactile hyper-reactivity explained 24% of the variance in total sleep disturbance scores in autistic children. Jamioł-Milc et al. (2021) investigated tactile modulation disorders (either hyper- or hypo-reactivity) and poor-quality sleep in autistic children using their own tools. While they did not find significant relationships because of small sample size, they suggested that insomnia in autistic children could be related to tactile hypo-reactivity. Ornitz et al. (1973) investigated auditory and vestibular input and the relationship to aspects of REM sleep. These investigators found fewer REM burst eye movements in the autistic children who slept on a custom-made mattress that delivered continuous sinusoidal vestibular input, compared to the non-autistic group. One suggested conclusion from this study was that the vestibular system might be hypo-responsive during sleep in young autistic children.

Hohn et al. (2019) examined the relationship between sleep concerns, measured using the Insomnia Severity Index (ISI; Morin, 1993), and sensory processing differences, using the Sensory Perception Quotient (SPQ; Tavassoli et al., 2014a) in autistic adults, along with a link to social skills. They predicted that findings would parallel those in autistic children, and might vary relative to the sensory domain addressed. Overall, they found subthreshold insomnia in their population, although it was higher than that in a neurotypical population. The severity of insomnia was predicted, and potentially driven by, high levels of visual reactivity (neither proprioception nor vestibular modulation were assessed), but also related to increased difficulty engaging in neurotypical social skills as measured by the Autism Spectrum Quotient short form (ASQ; Hoekstra et al., 2011). Sacco et al. (2010) included adults and children in their study, seeking to define factors that contribute to *autistic traits* using statistical methods. Using a purpose-built survey based on clinical *features of autism*, participant and family features, and supporting assessments, these investigators completed a complex principal components analysis resulting in the identification of four components that they hypothesized may allow for categorization of autistic endophenotype subgroups with some homogeneity. One such component was, “circadian and sensory dysfunction” which was linked to sleep disorders, self-injurious behavior, hyperactivity, decreased pain sensitivity, and differences and delays in language development. Authors suggest that sleep challenges and sensory differences could be connected in a complex manner.

### Effect of Sensory-Based Interventions on Sleep

We found relatively few studies we considered *sensory-based*, having a primary sensory intervention focus. Gee and colleagues (Gee et al., 2016, 2021a, 2021b^5^), as well as Gringras et al. (2014), investigated the use of weighted blankets in improving sleep for autistic children. Based on existing theory, they reasoned that the provision of deep touch pressure might release endorphins and serotonin, leading to a sense of calm. In each of the investigative reports by Gee et al. (2021a,b) a single subject ABA design with replication was used (*N* = 2 in each study). These investigators collected data across a 9-day baseline, 14-day intervention, and 7-day intervention withdrawal period in all studies. Outcomes reflected no meaningful improvement in CSHQ (33) sleep quality either subjectively or objectively. Gringras et al. (2014) conducted a more rigorous randomized control study with crossover, and a much larger sample size (*N* = 73). Based on the actigraph sleep measure used, the weighted blanket (used for 12–16 days) failed to lead to improved quantitative sleep scores, although parents perceived their child slept better and was calmer after using the weighted blanket. In addition, parents reported that their child liked sleeping under the weighted blanket.

Lawson and Little (2017) investigated a sensory-enhanced swim program for autistic children. In this pre/post single group study autistic children with sensory integration/processing concerns identified using the SP, participated in 8 weekly 1:1 swim lessons, each 30 min in length. There was also the opportunity for social interaction. The sensory enhancements to swimming were based on sensory strengths and needs, as identified on the sensory profile, along with visual schedules, communication strategies, physical supports, and modeling. They found the intervention to be feasible, with high parent satisfaction. Looking at sleep outcomes reflected on the CSHQ (33), 40% of the children showed reduced sleep disturbance, and 50% showed increased sleep disturbance. Of the 40% showing improvements in sleep, children were older, *autism severity scores* were lower, the children attended more swim sessions, and baseline sensory differences reflected sensory hyper-reactivity but low sensory seeking. Investigators concluded that children with sensory hyper-reactivity may be the best candidates for this intervention; they hypothesized that engagement in the intervention provided proprioceptive and tactile inputs that helped the children regulate their arousal. Johnson et al. (2021) also utilized a swimming intervention, provided in 12-sessions over a 3-week timeframe. Although the focus of this study was on child *challenging behavior* and parent wellbeing, parent report indicated improved sleep on the days of swim lessons.

Cullen et al. (2005) trained parents to implement a touch therapy (massage) program, titled “Training and Support Programme” (TSP). Parents received 8 weekly training sessions with the therapist and their child. In addition, they received written information which included instructions, diagrams, and photographs to guide the touch interactions with their child. Parents completed home record sheets and engaged in an interview 16 weeks from baseline. The home record sheets indicated that touch therapy sessions lead to calm, relaxation, and sleepiness in five of seven children, and improved sleep patterns for six of the seven children reported to have sleep difficulties. Other benefits were also noted, including an increased feeling of closeness between parent and child.

Silva et al. (2007) examined the effect of providing Cignolini Qigong massage to autistic children, determining if they could replicate outcomes from an earlier case series (Silva and Cignolini, 2005). Using a two-group design (treatment and control), trained practitioners initially provided massage (11 massage movements delivered in approximately 15 min) twice weekly for 5 weeks. Parents were given written and verbal instruction during initial sessions and demonstrated their ability to provide intervention during later sessions. For the next 5 weeks, the parent provided the massage at least once daily. Practitioner and parent interventions then alternated in 5-week blocks for the 5-month intervention. At the 5-month time, investigators offered intervention to the control group, based on improvements in the intervention group. Silva et al. (2007) reported global improvement in sensory processing scores on the *Sensory Profile* following the intervention, and a worsening of sensory concerns in the control group prior to being switched to intervention. Sleep improvements were reported by parents in areas including going to sleep at a typical time, faster sleep onset time and sleeping through the night.

## Discussion

While there is a body of literature addressing the sensory differences experienced by autistic individuals (cf. Crane et al., 2009; Elwin et al., 2017; Feldman et al., 2020) and another addressing sleep concerns (cf. Malow et al., 2006; Goldman et al., 2012; Morgan et al., 2020), the interplay between these constructs has not received the same degree of consideration. We report evidence indicating, at minimum, a co-existence of sensory integration/processing differences and sleep concerns in autistic children, and to some extent, autistic adults (Nieminen-von Wendt et al., 2005; Klintwall et al., 2011; Reynolds et al., 2012; Silva and Schalock, 2012; Tzischinsky et al., 2018; Wang et al., 2019; Eyuboglu and Eyuboglu, 2020; Kosaka et al., 2021). In some instances the relationship was predictive (Hohn et al., 2019; Mazurek et al., 2019). Some narrative reviews, however, have suggested a causal relationship between sensory sensitivity and difficulties with sleep in autism (cf. Cortesi et al., 2010; Reynolds and Malow, 2011). A handful of investigators have begun to examine interventions that are sensory-based or sensory incidental in nature, with some having a positive, or partially positive, impact on sleep (Cullen et al., 2005; Silva and Schalock, 2012; Lawson and Little, 2017). However, there are few intervention studies, and they are hampered by small sample sizes and often no comparison groups.

Of note, the vast majority of literature we identified was on children and teens; only Hohn et al. (2019) focused on adults, and results indicated that insomnia in autistic adults was predicted by high levels of sensory reactivity differences, along with decreased social skills as defined in neurotypical individuals, as measured by the ASQ. Approaching this data dimensionally—per the RDoC framework—Hohn and colleagues suggest that these relations indicate a cyclical influence between quality of sleep, sensory responsivity and the resources autistic adults have available to navigate neurotypical social interactions.

While Sacco et al. (2010) included adults in their sample, the mean age in this study was 8.82 ± 5.62 years. They did identify a component of autism characterized by circadian and sensory integration/processing differences, linking sensory integration/processing differences and sleep concerns across several life stages. Thus, while we have some insight into the relationship between sleep concerns and sensory integration/processing differences in autistic adults, this connection requires further investigation.

Sleep concerns in autistics run the full gamut; bedtime resistance, sleep onset delay, short sleep duration, sleep anxiety, night wakings, sleep disorder breathing, parasomnias, daytime sleepiness; and shortened total sleep have all been reported. Investigators have variably found that sleep in autistics may or may not be influenced by age (Tzischinsky et al., 2018), *autism severity* (Hollway and Aman, 2011), behavioral differences (Hollway and Aman, 2011), medications, (Hollway et al., 2013; Tzischinsky et al., 2018) and intellectual ability (Hollway et al., 2013). Similarly, sensory integration/processing differences encompass a range of findings, including hyper- and hypo-reactivity and sensory seeking, with investigators also reporting a variety of specific sensory domain differences. The outcomes related to specific sensory domains are somewhat conflicting. Hohn et al. (2019) suggested that visual sensory sensitivity is a driver for insomnia in autistic adults. In children, investigators report poor auditory filtering and taste/smell differences (Hollway et al., 2013), tactile hypo-reactivity (Wang et al., 2019; Jamioł-Milc et al., 2021), vestibular and oral hyper-reactivity (Kosaka et al., 2021), vestibular hypo-reactivity (Ornitz et al., 1973; Wang et al., 2019), and touch and oral hyper-reactivity (Tzischinsky et al., 2018).

As might be expected, the relationship between poor sleep and sensory integration/processing differences is often described as complex and multi-faceted. The inconsistencies noted by these investigators are likely related, in part, to the assessment and outcome tools used. As noted, investigators often used the CSHQ or a version of it. Other investigators used sleep diaries, or purpose-built tools, all relying on parent report. While literature reports consistency between parent report and objective measures of some domains of sleep (Malow et al., 2006), other sleep concerns may be under-estimated (e.g., night wakings) or over-estimated (e.g., total sleep duration) (Goodwin et al., 2007) by parents. The intervention study conducted by Gringras et al. (2014), and the characterization study by Kosaka et al. (2021) coupled parent report tools with an objective measure of sleep such as actigraphy. To optimally measure sleep challenges many investigators recommended the use of actigraphy, polysomnography or activity trackers to enhance accuracy.

A variety of frameworks and models have been proposed to describe sensory reactivity differences, and, while there is some overlap between the models, there is no current consensus regarding typology. Most of the studies examined in this review relied on the model derived from the Sensory Profile (SP; Dunn, 1999), the Short Sensory Profile (SSP; McIntosh et al., 1999), and the Sensory Profile 2 (Dunn, 2014). As noted earlier, in this model Dunn considers the interface between neurological threshold and self-regulation in response to sensation, and identifies patterns across four quadrants (poor sensory registration, sensory seeking, sensory avoiding, and sensory sensitivity). Reactivity differences within each sensory domain (sensory section scores) can be identified along a continuum from hyper- to hypo-reactivity, and within behavioral domains (behavioral section scores). Other models of sensory processing differences have also been developed, but few were used in the studies included in our review. Thus, our review findings regarding sensory processing differences are informed more by the SP, or a derivative of it, than any other model or tool. Furthermore, the selection of these tools emphasizes differences in sensory modulation and omits sensory discrimination and the sensory-based motor differences of posture and motor planning defined in a recent model of sensory integration (Bundy and Lane, 2020). Expansion of research to include these dimensions would enable investigators to explore the dynamic interactions among constructs that contribute to health and illness.

Sleep is a universal and core occupation throughout the lifespan. Inadequate sleep can interfere with synaptic development and brain maturation, attention, memory, mood regulation, behavior, and other aspects of daytime function (Killgore, 2010; Beebe, 2011), leading to restrictions in occupation and participation. While sleep difficulties are not uncommon in the neurotypical population, sleep disorders have been reported in up to 80% of autistic individuals (Richdale and Schreck, 2009; Souders et al., 2009; Cortesi et al., 2010; Morgan et al., 2020). Souders and colleagues suggested that autism alone may predispose individuals to sleep problems. Alternatively, being autistic in a neurotypical world may itself predispose individuals to sleep problems.

Differences in neural synaptic pruning and neural organization, circadian function, and melatonin production, and arousal and sensory processing have been identified in autistic individuals, and are putative causes of insomnia. The potential connection with sensory integration/processing and establishing supportive circadian rhythm cannot be overlooked. Verhoeff et al. (2018) suggest that sleep concerns are part of the overall picture of autism. They indicate that sleep problems in autistics increase as children age, and contrast this with the decrease in sleep problems across ages seen in the neurotypical population. The effect of sleep deprivation, the high incidence of sleep concerns in autistics, and the possibility that sleep concerns may worsen during childhood, make it critical to consider effective interventions. In addition, and importantly, sleep challenges for autistic children also have a negative impact on the sleep of their parents (Lopez-Wagner et al., 2008).

Therapeutic supports have been suggested. A recent review and meta-synthesis of sleep interventions for autistic children indicated that intervention fell into five broad approaches: pharmacological, melatonin (which is sometimes included in the pharmacological category), behavioral, parent education, and alternative therapies (Cuomo et al., 2017). In terms of pharmacological interventions, melatonin appears to have the strongest level of support, especially for sleep duration and latency to sleep onset. A major drawback relative to melatonin is that it may stop working after its initial success (Bruni et al., 2007). In addition, Esposito et al. (2020) indicated that parents of autistic children often prefer non-pharmacological interventions.

There is some evidence suggesting that behavioral interventions, which include a wide array of approaches ranging from extinction, to developing sleep hygiene, may have positive effects. Practice Guidelines from the American Academy of Neurology (Buckley et al., 2020) support trying behavioral interventions initially, and offering melatonin as a second line of defense. Esposito et al. (2020) suggested that behavioral interventions will not be a good fit for all families; such interventions should be guided by a knowledgeable clinician and utilized by motivated caregivers. They further indicated that approaches such as sleep hygiene and behavioral interventions are not well utilized or understood by parents, and are not well researched.

Parent education programs show some effectiveness, although it appears to be relatively weak (Cuomo et al., 2017). The Autism Treatment Network^6^ provides a parent guide and sleep tool kit for parents that addresses sleep setting and aspects of sleep hygiene; it also includes a supplemental calming module, addressing internal factors including sensory and arousal dysregulation that threaten sleep. However, Adkins et al. (2012) have suggested that written material alone is not sufficient support for parents. Both Cullen et al. (2005) and Silva et al. (2007) provided written materials and one-to-one training in their investigations of different forms of massage. Cullen and colleagues noted that, while some of the child participants initially found touch therapy (massage) unsettling, the majority accommodated over the course of the program. Cullen et al. (2005) indicated that parents reported positive changes in many behaviors; of the seven children with sleep concerns, six showed improvements in sleep. This investigation did not include a comparison group. Silva and colleagues also report improved sleep following a Cignolini Qigong intervention, along with positive changes in sensory processing, adaptive and social skills. While still a relatively small study (total *n* = 15), a comparison group was included, adding some rigor.

Alternative interventions, including types of massage described above, have been noted to have some positive outcomes. In conducting this review we identified studies that we interpreted as *sensory incidental*, delivering sensation as an important component of the intervention. Two were yoga-based interventions, which would have incorporated proprioception and vestibular inputs as well as addressing sensorimotor differences like posture and bilateral coordination. In a pilot and follow-up study Narasingharao et al. (2017a,b) provided yoga to autistic children, delivered by a trained yoga teacher; parents were encouraged to practice asanas at home with their child. Investigators documented changes in all aspects of sleep measured by a purpose-built questionnaire in both studies; parent report indicated uninterrupted and longer duration nighttime sleep, and a reduction in daytime sleeping. Sleep disordered breathing also improved. Sensory changes noted included better body balance and body awareness, although there was no clear measure of sensory differences. Frazier et al. (2017) examined the effectiveness and tolerability of the “Sleep-to-Sound” mattress. This mattress technology allows users to hear any audio file, feel vibration, or have both stimuli coming through the mattress; the intensity of both sensations could be set by the user. In this study, a baseline period was followed by a 2-week intervention (mattress either on or off) with immediate crossover. Findings indicated overall tolerability, and improvement in sleep parameters including sleep duration and efficiency, as determined by actigraphy. In addition, parent-completed sleep diaries indicated improved sleep quality and ease of falling asleep. Unfortunately, because neither of these studies included a measure of sensory integration/processing they were not included in our review. Further, Cuomo et al. (2017) point out that the available studies using alternative interventions are of low quality. The guidelines from the American Academy of Neurology (Buckley et al., 2020) indicate that there is no evidence for the efficacy of interventions such as weighted blankets or specialized mattresses, much as we reported in this review.

Overall, the range of potential interventions coupled with limited research to support most described interventions points to a large gap in existing literature. In fact, Cuomo and colleagues concluded that of the available interventions, no single approach was effective across all domains of sleep concerns. Further, noted interventions failed to adequately address pre-sleep concerns (e.g., sleep anxiety). Given the clear relation between sleep concerns and sensory integration/processing differences (including modulation, discrimination and sensorimotor dimensions) identified in this review, we suggest that future investigations consider the inclusion of tools to address sensory differences within sleep focused interventions may provide a missing piece to the sleep intervention puzzle. This is supported in a case report by Souders et al. (2017), in which pre-sleep calming was based on sensory needs, along with anxiety, interests, and preferences, with positive outcomes for both sleep and anxiety.

## Limitations

We limited our search to articles published in English and Spanish. As such we may have missed pertinent articles. In addition, there is considerable variability in terminology around sensory integration/processing; we may have missed some articles due to variability in keyword use. We did not restrict our review to studies with high rigor, thus some of the reported findings require replication. Some studies lacked detail, and this along with the variability in assessment tools and terminology made it challenging to summarize across studies.

## Conclusion

There is an established relationship between sleep concerns and sensory integration/processing differences in autistic children. In contrast, there is insufficient evidence to make this determination for autistic adults. Overall, research examining sensory and sleep differences in autistic individuals warrants future investigation. While the number of studies examining characteristics of this relationship exceeded those examining intervention, there is still much that is unclear. Intervention studies are few in number, and generally of low quality. This is an area ripe for future research.

## Author Contributions

SJL participated in developing search terms, initial abstract and title review, full-text review, and data extraction, was responsible for writing the initial draft, doing, and overseeing edits, and was first and primary author of the manuscript. MAL participated in developing search terms, running the search, de-duplicating, initial abstract, title review, full text review, and data extraction and assisted in manuscript development and editing. VS participated in full text review and data extraction, and contributed to the editing process. All authors contributed to the article and approved the submitted version.

## Conflict of Interest

The authors declare that the research was conducted in the absence of any commercial or financial relationships that could be construed as a potential conflict of interest.

## Publisher’s Note

All claims expressed in this article are solely those of the authors and do not necessarily represent those of their affiliated organizations, or those of the publisher, the editors and the reviewers. Any product that may be evaluated in this article, or claim that may be made by its manufacturer, is not guaranteed or endorsed by the publisher.

## Footnotes

1 We have chosen to use identity first language throughout this manuscript, in keeping with current trends in understanding autism.

2 What have historically been considered as *features, core features, core characteristics*, and *challenging behaviors* associated with autism are feasibly symptoms of autistic distress, vs. stable and perceptible differences (Lafrance and McKenzie-Mohr, 2013). We recognize that attributes such as *autistic features, core characteristics*, and *DSM-V diagnostic criteria for autism*, are hotly debated, particularly in context of recent participatory research (Sweet and Decoteau, 2017; O’Reilly et al., 2020). To honor current trends in understanding autism and in respecting autistic individuals, in this paper we italicize attributed terms and use identity first language.

3 National Institute of Mental Health, Research Domain Criteria Initiative (NIMH RDoC). https://www.nimh.nih.gov/research/research-funded-by-nimh/rdoc.

4 NIMH RDoC.

5 Gee et al., 2021a,b appear to use the same population and have the same aims. They have been treated as a single manuscript for reporting purposes.

6 Autism Treatment Network (n.d.). *A parents guide to improving sleep in children with autism*. https://www.autismspeaks.org/sites/default/files/2018-08/Sleep%20Tool%20Kit.pdf.
